# The genetic landscape of autism spectrum disorder in the Middle Eastern population

**DOI:** 10.3389/fgene.2024.1363849

**Published:** 2024-03-20

**Authors:** Yasser Al-Sarraj, Rowaida Z. Taha, Eman Al-Dous, Dina Ahram, Somayyeh Abbasi, Eman Abuazab, Hibah Shaath, Wesal Habbab, Khaoula Errafii‬, Yosra Bejaoui, Maryam AlMotawa, Namat Khattab, Yasmin Abu Aqel, Karim E. Shalaby, Amina Al-Ansari, Marios Kambouris, Adel Abouzohri, Iman Ghazal, Mohammed Tolfat, Fouad Alshaban, Hatem El-Shanti, Omar M. E. Albagha

**Affiliations:** ^1^ College of Health and Life Sciences, Hamad Bin Khalifa University, Doha, Qatar; ^2^ Qatar Biomedical Research Institute (QBRI), Hamad Bin Khalifa University, Doha, Qatar; ^3^ Qatar Genome Program, Qatar Foundation Research, Development and Innovation, Qatar Foundation, Doha, Qatar; ^4^ Quest Diagnostics Nichols Institute, San Juan Capistrano, CA, United States; ^5^ Pathology & Laboratory Medicine Department, Genetics Division, Sidra Medicine, Doha, Qatar; ^6^ The Shafallah Center for Children with Special Needs, Doha, Qatar; ^7^ Department of Pediatrics, Carver College of Medicine, University of Iowa, Iowa City, IA, United States

**Keywords:** autism spectrum disorder (ASD), neurodevelopmental disorders, epilepsy, next-generation sequencing (NGS), copy number variation (CNV), *de novo* mutation, genetics

## Abstract

**Introduction:** Autism spectrum disorder (ASD) is characterized by aberrations in social interaction and communication associated with repetitive behaviors and interests, with strong clinical heterogeneity. Genetic factors play an important role in ASD, but about 75% of ASD cases have an undetermined genetic risk.

**Methods:** We extensively investigated an ASD cohort made of 102 families from the Middle Eastern population of Qatar. First, we investigated the copy number variations (CNV) contribution using genome-wide SNP arrays. Next, we employed Next Generation Sequencing (NGS) to identify *de novo* or inherited variants contributing to the ASD etiology and its associated comorbid conditions in families with complete trios (affected child and the parents).

**Results:** Our analysis revealed 16 CNV regions located in genomic regions implicated in ASD. The analysis of the 88 ASD cases identified 41 genes in 39 ASD subjects with de novo (n = 24) or inherited variants (n = 22). We identified three novel *de novo* variants in new candidate genes for ASD (*DTX4*, *ARMC6*, and *B3GNT3*). Also, we have identified 15 *de novo* variants in genes that were previously implicated in ASD or related neurodevelopmental disorders (*PHF21A*, *WASF1*, *TCF20*, *DEAF1*, *MED13*, *CREBBP*, *KDM6B,* *SMURF1*, *ADNP*, *CACNA1G*, *MYT1L*, *KIF13B*, *GRIA2*, *CHM*, and *KCNK9*). Additionally, we defined eight novel recessive variants (*RYR2*, *DNAH3*, *TSPYL2*, *UPF3B KDM5C*, *LYST*, and *WNK3*), four of which were X-linked.

**Conclusion:** Despite the ASD multifactorial etiology that hinders ASD genetic risk discovery, the number of identified novel or known putative ASD genetic variants was appreciable. Nevertheless, this study represents the first comprehensive characterization of ASD genetic risk in Qatar's Middle Eastern population.

## 1 Introduction

Autism spectrum disorder (ASD) is a neurodevelopmental disorder distinguished by irregular social interaction and communication associated with repetitive behaviors and interests (Marini et al., 2020). ASD can be associated with different comorbidities, such as epilepsy, intellectual disability (ID), and attention deficit hyperactivity disorder (ADHD) (Freitag, 2007; Hyman et al., 2020). The worldwide prevalence of ASD is estimated to be approximately 1%, affecting males about four times more frequently than females (Levy et al., 2009; Hyman et al., 2020). Although there are ASD studies published from the Middle East (Amr, 2012; Alallawi et al., 2020; Yousef et al., 2021), the number is significantly disproportionate to the magnitude of the problem. Recently, a cross-sectional study of Qatari children aged 6–11 years surveyed between 2015 and 2018 revealed an ASD prevalence of 1.14% (95% CI: 0.89–1.46) (Alshaban et al., 2019). The Diagnostic and Statistical Manual of Mental Disorders (DSM-V) identifies ASD to include all social communication/interaction impairments and a minimum of two criteria in the restricted and repetitive behaviors (American Psychiatric Association, 2013).

ASD is one of the most heritable neuropsychiatric disorders, where the estimated recurrence rate is 5%–36% (Ozonoff et al., 2011; Werling and Geschwind, 2015). The high contribution of genetic factors to the etiology of ASD is suggested by a concordance rate of 60%–92% among monozygotic twins compared to 0%–10% among dizygotic twins (Bailey et al., 1995). Nevertheless, the recurrence rates among siblings born after two children affected by ASD per family revealed higher recurrence in males (47.5%) than in females (21.1%) and in siblings of females (44.3%) *versus* siblings of male probands (30.4%) (Werling and Geschwind, 2015).

ASD is clinically and genetically heterogeneous with several known monogenic disorders presenting with ASD symptoms, such as Fragile X syndrome (*FMR1*), Tuberous Sclerosis (*TSC1*, *TSC2*), and Rett syndrome (*MECP2*), contributing 1%–5% to the ASD etiology (Betancur and Coleman, 2013). Moreover, high throughput genetic testing identified genetic defects in ∼25% of the ASD cases (Yoo, 2015). These include genes involved in the synaptic formation, remodeling, and maintenance (*NRX1*, *CNTN4*, *DCLK2*, *CNT- NAP2*, *TRIM32*, *ASTN2*, *CTNTN5*, *SYN1*), neurotransmission (*SYNGAP1*, *GABRG1*, *CHRNA7*), and DNA methylation and chromatin remodeling (*MBD5*) (Lo-Castro and Curatolo, 2014). Pathogenic variants were primarily recurrent in *TSC1*, *TSC2*, *NF1*, *UBE3A*, and *MECP2* (Betancur, 2011). The vast majority of known ASD genes have a high incidence of *de novo* pathogenic variants. The accumulating number of distinct rare genetic causes of ASD (Durand et al., 2007; Morrow et al., 2008; Glessner et al., 2009; Wang et al., 2009) suggests a complex and heterogenous genetic architecture, similar to that of intellectual impairment and epilepsy (Jiang et al., 2004).

The genome-wide microarray studies in ASD patients have reported rare microdeletions or microduplications, collectively called copy number variation (CNV), that contribute to the increased risk of ASD and its associated comorbidities (Marshall et al., 2008; Sanders et al., 2011). Several studies of *de novo* events confirmed multiple CNVs in a considerable fraction (3%–20%) of ASD cases (Christian et al., 2008; Marshall et al., 2008; Pinto et al., 2010; Sanders et al., 2012). It has now been demonstrated that CNVs explained 3%–20% of the ASD genetic etiology over the past 5 years (Roberts et al., 2014; Zarrei et al., 2019; Yap et al., 2021). Our group described 3 CNV regions that were located in genomic regions implicated in ASD. We reported a patient with distal trisomy 10q syndrome presenting with a few previously undescribed physical features, as well as ASD (Al-Sarraj et al., 2014). Also, we described a case report of a male patient affected by ASD with a mosaic trisomy of the pericentromeric region of chromosome 8 and maternal uniparental disomy of the same chromosome (Ahram et al., 2016). Besides, we reported an ASD female patient with intellectual disability (ID) and epilepsy presenting with recurrent microdeletion [del(15) q24.1-q24.2]) (Ahram et al., 2017).

The genome-wide association studies (GWAS) of ASD conducted over the past 15 years were primarily performed on European populations (Ma et al., 2009; Wang et al., 2009; Weiss et al., 2009; Anney et al., 2010; Anney et al., 2012; Connolly et al., 2013; Autism Spectrum Disorders Working Group of The Genomics Consortium, 2017; Grove et al., 2019), and a few were on the Chinese and Korean populations (Cho et al., 2011; Kuo et al., 2015; Xia et al., 2020). Our group reported on GWAS of ASD in the Middle Eastern population of Qatar using a family-based approach (Al-Sarraj et al., 2021). Results showed that common single nucleotide polymorphisms (SNP) are associated with ASD. Although the identified loci did not reach genome-wide significance, many of the top associated SNPs are located within or near genes that have been implicated in ASD or related neurodevelopmental disorders (Al-Sarraj et al., 2021).

There is a rapid rise in studies utilizing genomic sequencing to identify ASD genetic risk factors (Chapman et al., 2015; Yuen et al., 2015; Satterstrom et al., 2020; Kim et al., 2021). Most of these studies investigated and identified rare *de novo* variants in multiple genes, such as *SCN2A*, *CHD8, ADNP*, *SHANK3*, *PTEN*, *DEAF1*, and *ANKRD11* (Yuen et al., 2015; Turner et al., 2017; Satterstrom et al., 2020). Lately, there has been a striking increase in the studies aimed at identifying ASD susceptibility genes in multiple populations using genomic sequencing (Besenbacher et al., 2015; Ye et al., 2015; Chang et al., 2019; Tran et al., 2020; Kim et al., 2021). Nevertheless, improved genomic techniques and analyses have expedited the detection of genes involved in ASD (Shen et al., 2014).

In this study, we inspected the contribution of CNVs in 113 patients with ASD, then we investigated the mutational spectrum of ASD patients using whole exome/genome sequencing in the families of 88 ASD cases. This study represents the first comprehensive genetic study of ASD in Qatar’s Middle Eastern population using simplex (trio) and complex families (families with multiple affected individuals).

## 2 Results

### 2.1 Characteristics of the study population

**FIGURE 1:**
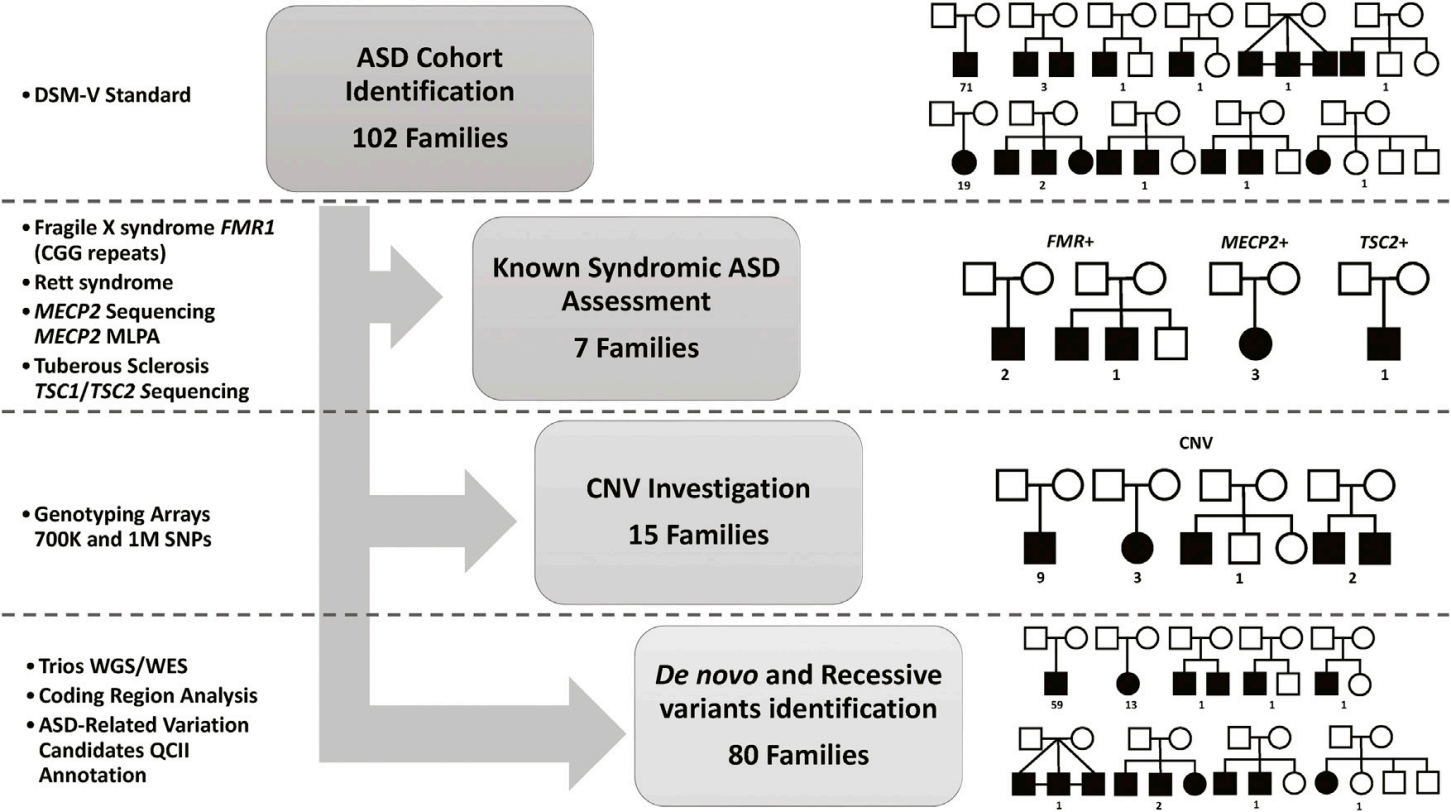
Summary of the Study Cohort and Analysis Approach. DSM-V, the Diagnostic and Statistical Manual of Mental Disorders standards for ASD identification. Fragile X syndrome (Genotyping for CGG expansion in *FMR1*), Rett syndrome (Sanger Sequencing for *MECP2* mutations identification or microdeletion identifications using Multiplex Ligation Dependent Probe Amplification (MLPA)), and Tuberous Sclerosis (Sanger Sequencing for *TSC1* or *TSC2* mutations identification). Single Nucleotide Polymorphisms (SNP)) markers for Copy Number Variation (CNV) identification. Whole-genome Sequencing (WGS) and Whole-Exome Sequencing (WES) are used for *de novo* and recessive mutations identification in all trios (both parents and affected children), as well as other affected or healthy siblings for multiplex families. QIAGEN Clinical Insight Interpret (QCII) for variant interpretation and annotation.

The clinical characteristics of the study cohort are shown in Table 1. The study comprised 102 families, of whom 90 were simplex trios, 4 with at least one unaffected sibling, and 8 were multiplex families with more than one affected and/or unaffected sibling. All pedigrees of the study cohort and a summary of the work approach and studied individuals are described in Figure 1. The average age of ASD patients, mother, and father were 8.9, 36.5, and 42.1, respectively (Table 1). In addition, our cohort consisted of 24 ASD individuals who had Epilepsy as a comorbidity.

**TABLE 1:** Clinical characteristics of the ASD study cohort.

	ASD	Parent	Maternal	Paternal
Number of subjects	113	178	88	87
Age (mean SD±)	8.86 ± 10.5	39.4 ± 8.1	36.5 ± 6.4	42.1 ± 8.5
Male/Female Ratio	3.64:1	-	-	-
Epilepsy	24	-	-	-
Consanguinity	-	36^*^	-	-

*The reported consanguinity is for 36 pairs.

### 2.2 Molecular evaluation of individuals with ASD

We first investigated known syndromic causes of ASD in all identified ASD-affected individuals, namely, fragile X syndrome (caused by CGG expansion in *FMR1*), Rett syndrome (caused by mutations in *MECP2*), and Tuberous Sclerosis (caused by mutations in *TSC1* or *TSC2*), as explained in Figure 1. Analysis of the study cohort identified four male subjects with Fragile X syndrome in three families (the repeat length exceeded 200 CGG repeats for all cases), three female subjects with known pathogenic variants in *MECP2* (Rett syndrome), and one subject with Tuberous Sclerosis caused by c.5024 C>T/p.Pro1675Leu known pathogenic variant in *TSC2*. Next, we investigated the contribution of CNVs to ASD etiology in our cohort using SNP genotyping arrays to identify pathogenic CNVs related to ASD, as shown in Figure 1. Analysis of CNVs identified 16 CNVs (7 segmental loss and nine segmental gain) in 16 subjects from 15 families (Supplementary Table S1) that were reported in the DECIPHER database. To illustrate the CNVs identified in our ASD cohort, we compared and aligned examples of the identified CNV regions between the Proband, Mother, and Father for Case 8, Case 11, and Case 16 presented in (Supplementary Figure S1). Detailed information about each detected CNV is presented in Supplementary Table S1). Following the exclusion of classical known ASD disorders and potential CNV regions, our study focused on a cohort of 80 ASD nuclear families. This group consisted of 72 simplex families, each with one affected individual (n = 72 ASD affected), and eight multiplex families. Within the multiplex families, five had additional affected siblings (n = 13 ASD affected), while the remaining three included at least one unaffected sibling (n = 3 ASD affected). This cohort was then subjected to next-generation sequencing (NGS) analysis to identify *de novo* or novel mutations in genes implicated in the etiology of ASD. These families were evaluated using genome or exome sequencing to identify ASD-related mutations. SNP genotype data analysis identified 28 consanguineous and 52 non-consanguineous families. The male-to-female ratio was 4.2 to 1 (71 males and 17 females), which is consistent with data from previous studies (Yuen et al., 2015; Loomes et al., 2017; Alshaban et al., 2019; Geetha et al., 2019; Samia et al., 2020), and 25% of ASD cases had epilepsy as a comorbidity.

### 2.3 De novo variant identification in simplex and multiplex ASD families

Next, of the 88 ASD cases, we identified 24 with *de novo* variants in 22 subjects (Table 2). Two out of the 22 subjects were found to carry two *de novo* variants. Nevertheless, we identified 24 genes with *de novo* variants, 19 had established evidence or were predicted to be implicated in ASD, epilepsy, or intellectual disability. For instance, there were nine genes (*NLRP5* (Docherty et al., 2015), *DRD5* (Nguyen et al., 2014), *TCF20* (Vetrini et al., 2019), *DEAF1* (Satterstrom et al., 2020), *CREBBP* (Zheng et al., 2016), *KDM6B* (Stolerman et al., 2019), *ABCA2* (O’ Roak et al., 2012), *MYT1L* (Blanchet et al., 2017), and *CHRNG* (Kalınlı et al., 2019) that strongly associated with the Qiagen Clinical Insights (QCI) phenotype-driven ranking scores. Furthermore, we assessed the strength of evidence for our potential candidate identified ASD genes by comparing them with the SFARI database. Candidate genes reported in our patients with no reported evidence of ASD or neurodevelopmental disorders are shown in Supplementary Table S2.

**TABLE 2:** *De novo* variants in ASD patients detected in genes with substantial evidence of involvement with ASD.

Patient ID	Protein^*^	Gene	CADD score	dbSNP ID*	gnomAD frequency^**%^	QCI computed pathogenicity^$^	SFARI genes score^***^	ASD Evidence∀
3	p.K468T	***DTX4 NM_001300727.2***	28.10	NA	NA	VUS	NA	Zhou et al. (2022)
4	p.P320R	*NLRP5 NM_153447.4*	0.01	771929862	0.0004	VUS	NA	Docherty et al. (2015)
12	p.A131V	***ARMC6 NM_033415.4***	27.00	769059471	0.007	VUS	NA	NA
14	p.T469I	*DRD5 NM_000798.5*	22.5	NA	NA	VUS	NA	Nguyen et al. (2014)
16	p.P399Q	*PHF21A NM_001101802.3*	29.90	NA	NA	VUS	1	Kim et al. (2019)
19	p.R72H	*WASF1 NM_003931.3*	29.60	1037010853	0.001	VUS	S	Ito et al. (2018)
24	p.P1942R	*TCF20 NM_001378418.1*	23.70	144341537	0.09	VUS	1	Vetrini et al. (2019)
25	p.D251G	*DEAF1 NM_021008.4*	27.10	NA	NA	VUS	1	Rajab et al. (2015)
26	p.V346I	*MED13 NM_005121.3*	17.53	NA	NA	VUS	1	Snijders Blok et al. (2018)
27	c.870-10T>G	*SETD1A NM_014712.3*	15.07	750087609	0	VUS	1	Spataro et al. (2023)
27	p.H1432R	*CREBBP NM_001079846.1*	26.50	797044860	NA	Likely Pathogenic	1	Zheng et al. (2016)
28	p.S1198L	*KDM6B NM_001348716.2*	31.00	NA	NA	VUS	1	Stolerman et al. (2019)
34	p.G724R	*SMURF1 NM_181349.3*	29.90	956288312	0.003	VUS	2	De Rubeis et al. (2014)
35	p.D397G	*ABCA2 NM_001606.5*	21.4	NA	NA	VUS	NA	O’Roak et al. (2012)
36	p.Y719*	*ADNP NM_001282531.3*	22.80	587777526	0.002	Pathogenic	1	Helsmoortel et al. (2014)
38	p.E11*	*CACNA1G NM_198396.3*	36.00	NA	NA	VUS	2	Chemin et al. (2018)
38	p.A167V	***B3GNT3 NM_014256.4***	23.20	NA	NA	VUS	NA	Zhou et al. (2022)
40	p.V13A	*CHM NM_000390.4*	26.20	755235198	0.001	VUS	3	Tran et al. (2020)
43	p.G573R	*MYT1L NM_015025.4*	29.9	1330054460	NA	VUS	1	Blanchet et al. (2017)
45	p.R1086*	*KIF13B NM_015254.4*	39	NA	NA	VUS	2	Chen et al. (2020)
46	p.W580C	*GRIA2 NM_001379001.3*	26.1	NA	NA	VUS	1	Salpietro et al. (2019)
47	p.V160A	*KCNK9 NM_001282534.2*	25.7	NA	NA	VUS	NA	Delgado et al. (2014)
48	p.R458C	*CHRNG NM_005199.5*	23.40	762066089	0.003	VUS	NA	Kalınlı et al. (2019)
52	p.K337R	*KDM2A NM_001256405.2*	23.2	NA	NA	VUS	3	Iossifov et al. (2014)

Summary of 24 *de novo* variants detected in 22 patients with ASD., Identified variant(s) per family separated by borderline. Novel candidate genes are shown in bold font. Reported genes as the Reference Sequence (RefSeq) name and accession numbers.NA, indicates novel mutations not previously reported in the gnomAD or SFARI, databases. ** Reported gnomAD, frequency was observed out of 141,456 individuals. ^***^ SFARI, Gene Score is a ranking system that estimates the strength of evidence of the reported ASD, gene in the SFARI, database, with S = ASD, syndromic category and scores ranging from 1 to 3, with 1 being the higher score indicating the stronger association evidence with ASD. ^$^ The imputed ACMG, classification criteria used in QCI, Interpret; VUS, is a variant of uncertain significance. ∀ Genes have been previously associated with or reported in patients with ASD, or neurodevelopmental disorders.

In addition, to validate the *de novo* variants, stringent filtration criteria were implemented on the trio members (Proband, Mother, and Father) to eliminate variants with poor base and mapped quality or variants with low coverage and allele depth. Moreover, *de novo* variants were only considered if they had high-quality sequencing data from the proband, mother, and father (Supplementary Table S3). The two *de novo* variants, which were detected in the same ASD subject, and three additional randomly selected variants were tested for validation using Sanger sequencing (Supplementary Figures S2–S4).

**FIGURE 2:**
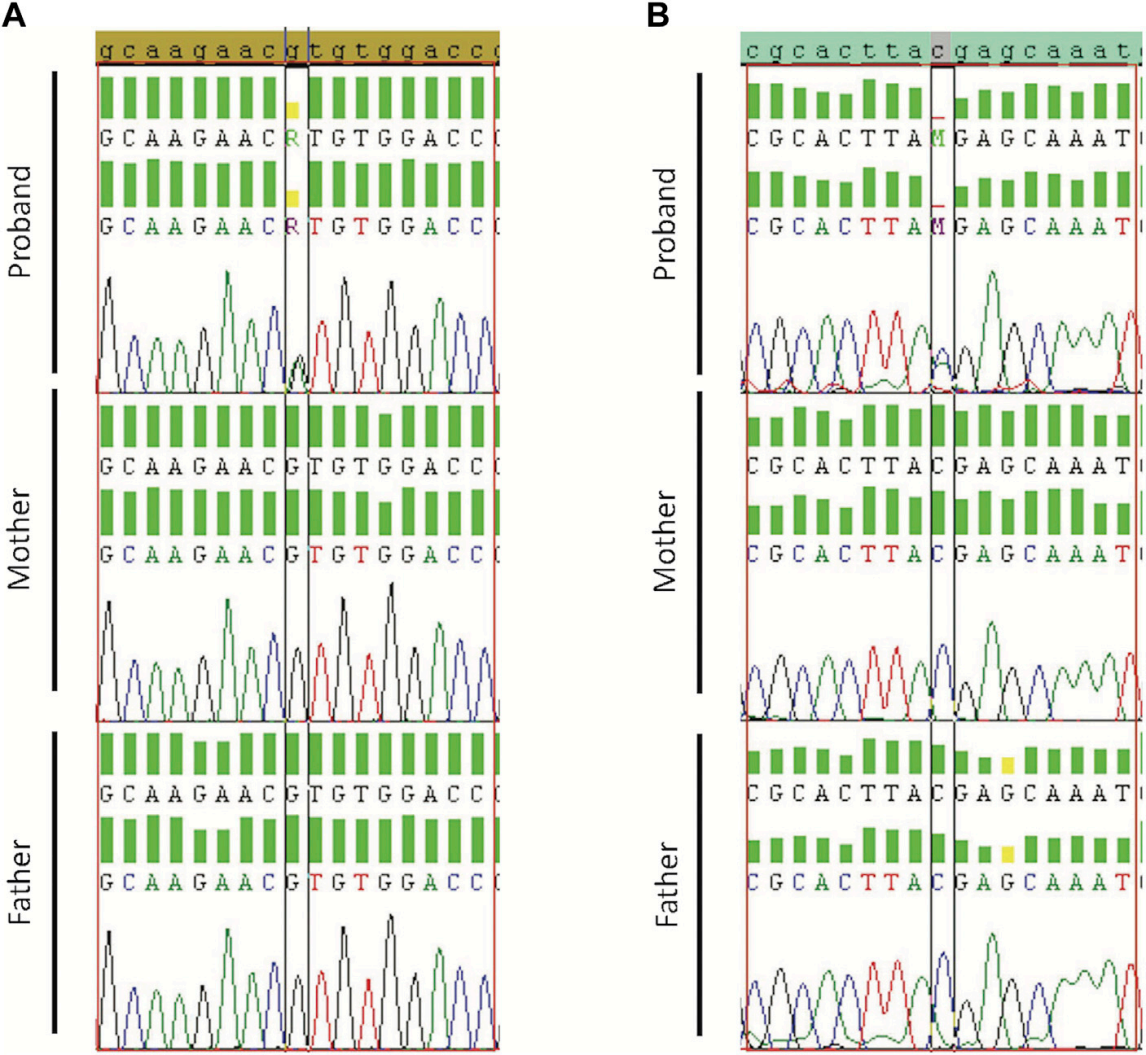
Sanger sequencing results for Proband, Mother, and Father. **(A)** Validation of *WASF1 de novo* variant for case19 with *WASF1* variant (c.215G>A/p.R72H). **(B)** Validation of *ADNP* for case 36 with *ADNP* variant (c.2157C>A/p.Y719*). Borders indicate *de novo* alteration (in the child) and wild-type allele (in parents).

Validated *de novo* variants showed the presence of the variant in the proband but not the parents. Sanger sequencing results confirmed all the five tested variants (Case19 with *WASF1* variant (c.215G>A/p.R72H) Figure 2A; case 36 with *ADNP* variant (c.2157C>A/p.Y719*) Figure 2B; and case34 with *SMURF1* variant (c.2170G>A/p.G724R) Supplementary Figure S2). Also, we confirmed the *de novo* variants in case 38, one in *CACNA1G* (c.31G>T/p.E11*) and a second in *B3GNT3* (c.500C>T/p.A167V) Supplementary Figures S3, S4.

### 2.4 Recessive variants in simplex and multiplex families with ASD

In parallel, ASD-affected individuals from consanguineous parents were processed for recessive and X-linked variant identification (Yu et al., 2013; Cukier et al., 2014). Hence, we identified 21 recessive variants (16 autosomal and 4 X-linked) in 19 ASD subjects (Table 3). Most detected variants were single nucleotide variants (SNVs; n = 17), but we also identified 2 Indels. Interestingly, about 16% of these variants were located in genes implicated in ASD, as revealed by the QIAGEN knowledge base shreds of evidence. Also, our analysis revealed five recessive variants described in genes with strong phenotype-driven ranking scores within the QCI. These include *VPS13B* (Yu et al., 2013), *CADPS2* (Grabowski et al., 2017), *LYST* (Manoli et al., 2010), *TECTA* (Toma et al., 2014), and *C12orf57* (Akizu et al., 2013).

**TABLE 3:** Recessive variants in ASD patients detected in genes with substantial evidence of involvement in ASD.

Patient ID	Protein	*Gene*	CADD score	dbSNP ID*	gnomAD frequency **^%^	gnomAD homozygous count	QCI computed pathogenicity^$^	SFARI genes score	ASD Evidence∀
1	p.R2814Q	*VPS13B NM_017890.5*	32	148333124	0.0002	0	VUS	1	Yu et al. (2013)
1	p.E122Q	*PIGN NM_012327.6*	26.7	200756305	0.07	1	VUS	NA	Maydan et al. (2011)
55	p.R2814Q	*VPS13B NM_017890.5*	32	148333124	0.0002	0	VUS	1	
55	p.E122Q	*PIGN NM_012327.6*	26.7	200756305	0.07	1	VUS	NA	
56	p.R2814Q	*VPS13B NM_017890.5*	32	148333124	0.0002	0	VUS	1	
56	p.E122Q	*PIGN NM_012327.6*	26.7	200756305	0.07	1	VUS	NA	
5	p.V1594F	*RYR2 NM_001035.3*	24.3	NA	NA	NA	VUS	NA	Hamdan et al. (2014)
6	p.V3189fs*7	*DNAH3 NM_017539.2*	<10	NA	NA	NA	VUS	2	Guo et al. (2017)
6	p.Q556H	*TSPYL2 NM_022117.4*	17.95	NA	NA	NA	VUS	NA	Moey et al. (2016)
57	p.Q556H	*TSPYL2 NM_022117.4*	17.95	NA	NA	NA	VUS	NA	
11	p.T2359N	*SYNE NM_182961.41*	22.8	142747430	0.014	0	VUS	2	Jiang et al. (2013)
11	p.Q107L	*TGIF1 NM_174886.3*	23.1	28939693	0.031	0	Likely Benign	NA	Latypova et al. (2021)
13	p.D267G	*CADPS2 NM_139175.2*	24.9	773840565	NA	NA	VUS	2	Okamoto et al. (2011), Grabowski et al. (2017)
15	p.F82L	*UPF3B NM_080632.3*	23.6	NA	NA	NA	VUS	1	Tejada et al. (2019)
17	p.R332W	*CHRNG* NM_005199.5	25.7	567899708	0.066	4	VUS	NA	Kalınlı et al. (2019)
18	p.V212A	*AMT NM_001164712.2*	27.1	201141125	0.0003	0	VUS	2	Yu et al. (2013)
21	p.A241fs	*KDM5C NM_001353981.2*	NA	NA	NA	NA	VUS	1	Moey et al. (2016)
22	p.A194T	*FH NM_000143.4*	29.9	587782215	0.006	0	VUS	NA	Luo et al. (2020)
30	p.R1060H	*ASTN1 NM_207108.3*	26	201593312	0.007	0	VUS	NA	Lionel et al. (2014)
31	p.N2868Y	*LYST NM_000081.4*	25.9	NA	NA	NA	VUS	NA	Manoli et al. (2010)
32	p.P537S	*EPHA6 NM_001080448.3*	26.8	1463599601	0.001	0	VUS	NA	Schneider et al. (2015)
32	p.R474C	*TECTA NM_005422.4*	27.6	756326790	0.002	0	VUS	2	Toma et al. (2014)
32	p.P1087S	*ROBO2 NM_002942.5*	21.6	0.00006409	0.00007	NA	VUS	2	Connolly et al. (2017)
47	p.G355E	*TRAF3IP1 NM_015650.4*	29.60	560016209	0.012	0	VUS	NA	Bizet et al. (2015)
49	p.M1V	*C12orf57 NM_001301834.1*	21.9	587776954	0.003	0	VUS	S	Akizu et al. (2013)
50	p.M1V	*C12orf57 NM_001301834.1*	21.9	587776954	0.003	0	VUS	S	
50	p.E79G	*WNK3 NM_020922.5*	22.9	NA	NA	NA	VUS	2	Qiao et al. (2008)

Summary of 21 recessive variants detected in 19 patients with ASD., Identified variant(s) per family separated by borderline. Reported genes as the Reference Sequence (RefSeq) name and accession numbers.NA, indicates novel variants not previously reported in the gnomAD or SFARI, databases. ** Reported gnomAD, frequency was observed out of 141,456 individuals. ^***^ SFARI, Gene Score is a ranking system that estimates the strength of evidence of the reported ASD, gene in the SFARI, database, with S = ASD, syndromic category and scores ranging from 1 to 3, with 1 being the higher score indicating the stronger association evidence with ASD. ^$^ The imputed ACMG, classification criteria used in QCI, Interpret; VUS, is a variant of uncertain significance. ∀ Genes have been previously associated with or reported in patients with ASD, or neurodevelopmental disorders.

On the other hand, all reported recessive variants were assessed for transmission in trio analysis, and only those showing transmission were considered. The identified variants were then annotated using several available databases, including Genome Aggregation Database (gnomAD) (Karczewski et al., 2020), Exome Aggregation Consortium (ExAC) on non-psychiatric disease samples (Walsh et al., 2017), and Greater Middle Eastern (GME) database (Scott et al., 2016) to identify potential novel findings. Additionally, we evaluated the strength of evidence for our potential ASD candidate genes by comparing them to the SFARI database. Genetic variations in candidate genes with no substantial association or evidence of ASD or neurodevelopmental disorders are shown in Supplementary Table S4. Finally, we assessed if any affected child carries both *de novo* and recessive variants in simplex and multiplex families with ASD. Of 88 ASD patients, we identified two affected individuals with *de novo* and inherited variants, as described in (Table 4).

**TABLE 4:** *De novo* and recessive variants in ASD patients detected in genes with substantial evidence of involvement in ASD.

Patient ID	Protein	Gene	CADD score	Mode of inheritance	dbSNP ID	gnomAD frequency^**%^	gnomAD homozygous count	SFARI genes score	ASD Evidence∀
34	p.G724R	*SMURF1 NM_181349.3*	29.90	*De Novo*	956288312	0.003	0	2	De Rubeis et al. (2014)
34	p.P280S	*NAALAD2 NM_001300930.2*	26.10	Recessive	200163147	0.012	0	NA	-
47	p.G355E	*TRAF3IP1 NM_015650.4*	29.60	Recessive	560016209	0.012	0	NA	Bizet et al. (2015)
47	p.V160A	*KCNK9 NM_001282534.2*	25.70	*De Novo*	NA	NA	NA	NA	Delgado et al. (2014)

Summary *de novo* and recessive variants identified in each patient with ASD., Identified variant(s) per family separated by borderline. Reported genes as the Reference Sequence (RefSeq) name and accession numbers. NA, indicates novel variants not previously reported in the gnomAD or SFARI, databases. ** Reported gnomAD, frequency was observed out of 141,456 individuals. ^***^ SFARI, Gene Score is a ranking system that estimates the strength of evidence of the reported ASD, gene in the SFARI, database, with S = ASD, syndromic category and scores ranging from 1 to 3, with 1 being the higher score indicating the stronger association evidence with ASD. ∀ Genes have been previously associated with or reported in patients with ASD, or neurodevelopmental disorders. ∀ Genes have been previously associated with or reported in patients with ASD, or neurodevelopmental disorders.

## 3 Methods

### 3.1 Study subjects

Three hundred and twenty-nine individuals from 91 simplex and 11 multiplex families were recruited for this study. Study subjects were recruited from the Shafallah Center for Children with Special Needs (Doha, Qatar). ASD diagnosis was ascertained by clinical geneticists according to the Diagnostic and Statistical Manual of Mental Disorders (DSM-V) standards, and the clinical phenotype was delineated in affected individuals, including ASD-associated comorbidities, congenital anomalies, dysmorphic features, the Autism Diagnostic Interview-Revised (ADI-R) and/or Autism Diagnostic Observation Schedule (ADOS), and Intelligence Quotient (IQ) measure when possible. A signed informed consent was obtained from the participants or their Legally Authorized Representatives. The study was approved by the Qatar Biomedical Research Institute’s Institutional Review Board (IRB).

### 3.2 DNA extraction

About 3 mL of whole blood samples was drawn, in EDTA tubes, from all available family members of the ASD proband. DNA was extracted with the Gentra-Puregene Blood kit from [Qiagen, Valencia, CA], according to the manufacturer’s specifications and protocols. Total DNA amount and purity were measured by NanoDrop 2,200 Spectrophotometers from (ThermoScientific) and Qubit dsDNA BR or HS assay kit and Qubit 2.0 system from [Invitrogen] according to the manufacturers’ guidelines.

### 3.3 Molecular assessment of known syndromic ASD

Fragile-X syndrome molecular testing was conducted by direct PCR of extracted DNA using primers for *FMR1* and fluorescent capillary electrophoresis of the amplified CGG-repeat to determine their number. Alleles above 120 CGGs are challenging to detect using this technique (Chen et al., 2010). If no *FMR1* allele is detected in males or a single normal allele is detected in females, the testing continued with a Southern blot analysis. *FMR1* was exposed to digestion with two endonuclease enzymes, one methylation-sensitive, followed by Southern hybridization and analysis of the produced fragment (Sofocleous et al., 2009).

The Rett syndrome molecular testing (*MECP2*) was performed by PCR amplification of extracted DNA followed by Sanger resequencing analysis of the entire coding region of *MECP2* and splice sites. Further testing by Multiplex Ligation dependent Probe Amplification (MLPA) (Erlandson et al., 2003) for deletions and duplications was performed for individuals who tested negative via sequencing.

The Tuberous Sclerosis Complex molecular diagnostic testing was performed by PCR of extracted DNA followed by Sanger resequencing using primers for the entire coding region of *TSC* and *TSC2* and splice sites (Milunsky et al., 2009).

### 3.4 SNP genotyping

SNP genotyping was conducted for all ASD probands and their family members using Illumina’s Infinium Bead Chip Human1M-Duo-v3 (1.119 million SNPs) or HumanOmniExpress-12v1-1 (719,000 SNPs) (Illumina, San Diego, CA) by following the manufacturer’s protocol. SNP genotype data were first analyzed using Genome Studio software followed by PLINK v1.9 (Chang et al., 2015) to perform quality control measures and assess consanguinity. Illumina GenomeStudio-2.0 plug-in was used for the copy number variations analysis by applying two independent algorithms, cnvPartition-v3.2.0 (Tsuang et al., 2010) and QuantiSNP-v2 (Colella et al., 2007).

### 3.5 Whole-exome and whole-genome sequencing

Exome sequencing library preparation was performed for 298 individuals using the SureSelect Human All Exon v6. The SureSelectXT target size is around 60 Mega Base (MB) which cover the genomic coding and UTRs regions and the splice sites boundaries (site of exon-intron boundaries). Exomes were sequenced on the HiSeq4000 utilizing the HiSeq3000/4000 SBS Kit 300 Cycles reagents. Whole Genome sequencing was performed for 63 samples (21 trios) by BGI genomics (n = 27), or CompleteGenomics (n = 36) (Drmanac et al., 2010).

### 3.6 Validation by Sanger sequencing

For identified *de novo* variant validation, primers were designed using the web-based tool Primer3web (https://primer3.ut.ee/). PCR-amplified targets were then subjected to Sanger sequencing using an automated sequencer (Applied Biosystems ABI-3700).

### 3.7 Data analysis approach for exome/genome sequencing

Data quality control assessment of the generated Fastq files was performed using FastQC (v0.11.5) to estimate quality metrics and evaluate the quality of the raw-sequenced reads. Analysis of sequence data generated from the Hiseq4000 system was done through in-house and optimized scripts using Applied Genome Analysis Tool Kit (GATK-4.0.11) best practices pipeline for short variants (SNPs and Indels). Sequencing reads data were aligned to human genome reference (hg19). PLINK v1.91 (Purcell et al., 2007) was employed to evaluate the quality of the generated variants for all samples (variants call rate, excess heterozygous, and gender mismatch). To assess consanguinity among the recruited families, we computed the inbreeding coefficient for parents using PLINK v1.9, where we determined a subset of independent autosomal SNPs (around 58K pruned SNPs, using a window size of 200 SNPs and LD threshold of r^2^ = 0.05).

Variants with Mendelian violation were estimated then eliminated by the GATK-4.011 FindMendelianViolation tool to exclude samples with evidence of contamination during the wet lab preparation.

For the genome sequencing performed at BGI and CompleteGenomics data, a list of variants was received for each genome in a standard variant call format (vcf) file. Nevertheless, the WGS data were subjected to the same QC and downstream analysis as the WES data.

The variant interpretation and annotation were processed by QIAGEN Clinical Insight Interpret (QCII) version 9.0 to identify significant variants in genes involved in neurological development as follows described below.

### 3.8 De novo variant identification

The first set of analyses involved the identification of *de novo* variants. The QCII-analysis setup and filtrations were initiated to keep all variants with passed upstream pipeline filtering, genotype quality ≥30, and read depth ≥10 in cases and controls. Also, variants outside the top 1% of most exonically variable genes in healthy public genomes (1,000 genomes) were included. Next, *de novo* variants were excluded for variants with an allele frequency ≥0.1% in the 1,000 genomes project, the NHLBI ESP exomes (All), ExAC, and the gnomAD databases. Variants were then filtered to keep only variants in exons and up to 20 bases into the intron with allele fraction of at least 35 in cases or controls. In this step, variants were annotated for their functional effect and disease-association (clinically relevant) using the QCII platform. The following keywords were used for phenotype: Autism, Autism spectrum disorder, epilepsy, intellectual disability, and mental retardation or combinations of these terms.

### 3.9 Recessive variant identification

Initially, we performed the recessive variant analysis in the same way as the first three steps applied in the QCII *de novo* analysis described above. However, we altered the genetic analysis (mode of inheritance) to keep homozygous or hemizygous variants in at least one of the case samples at the variant level. Simultaneously, these variants should not occur in any control samples (homozygous or hemizygous). Besides, the identified homozygous or hemizygous should be consistent with the inheritance transmitted by parents. Finally, the variants were annotated as described in the QCII *de novo* analysis using the same phenotype keywords.

### 3.10 QIAGEN Clinical Insight Interpret version 9.0.0.20220826 freeze

The variants reported correspond to the hg19 human genome reference sequence. The databases used in the variant annotation process are listed in Supplementary Table S5.

### 3.11 SFARI genes database 01-16-2024 release

To assess the strength of the evidence, we annotated the identified genes to the SFARI (Simons Foundation Autism Research Initiative) genes database, which is a comprehensive resource that catalogs genes potentially associated with ASD. Genes in the SFARI Gene database are assigned scores with S for syndromic ASD cases, then defined ranging score from 1 to 3, with 1 being the higher score indicating stronger evidence of association with ASD (Abrahams et al., 2013).

## 4 Discussion

We present a comprehensive study for ASD in the Middle Eastern population of Qatar using WGS/WES of simplex (trio) and multiplex families. We identified 40 candidate genes for ASD in 39 ASD subjects. Many of the identified genes had previously been implicated in ASD or related neurodevelopmental disorders. We identified three novel *de novo* variants in genes not previously linked to ASD (*DTX4*, *ARMC6*, and *B3GNT3*). All three *de novo* mutations were predicted to be damaging and rare, with either never reported in gnomAD and dbSNP or having allele frequencies less than 0.001%. We have defined these genes as highly conceivable suggested novel genes not reported in the SFARI database. Still, the literature showed that these genes are expressed in the human brain and indirectly illustrate a link to ASD or neurodevelopmental disorders (NDD). However, it necessitates future functional studies to ascertain their potential role in ASD or NDD pathogenesis.

Moreover, recent studies investigating exome or genome sequencing data aggregation of ASD cases have identified *de novo* mutations in *DTX4* and *B3GNT3* (Fu et al., 2022; Zhou et al., 2022). *DTX4* (Deltex E3 ubiquitin ligase 4) is mainly expressed in the brain and is denoted to be involved in the Notch signaling pathway and protein ubiquitination. *NLRP4* recruits *DTX4*, and through their interaction, it promotes the degradation of TBK1(Cui et al., 2012). Recent studies highlighted ubiquitin’s role in neurodevelopmental disorders and reported their monogenic forms to provoke lesions in gene coding, including ubiquitin proteins. These studies hypothesize a possible autoinflammation involvement in neurodevelopmental disorders pathogenesis (Ebstein et al., 2021). *ARMC6* (armadillo repeat containing 6), the function of this gene’s protein product has not been determined. However, this gene is expressed in the brain. *ARMC6* belongs to the Armadillo repeat-proteins (ARMCs) family which are distributed in eukaryotes and play prominent roles in cell-cell adhesion, intracellular signaling, and cytoskeletal regulation (Song et al., 2019). Also, *in situ* hybridization revealed a high expression of Armcx6 in mouse neuronal tissue in the developing and adult nervous system (López-Doménech et al., 2012). *B3GNT3* (UDP-GlcNAc:betaGal beta-1,3-N-acetylglucosaminyltransferase3) gene is involved in the biosynthesis of poly-N-acetyllactosamine chains and the biosynthesis of the backbone structure of dimeric sialyl Lewis a (Zhang et al., 2015). This gene is mainly expressed in the intestine, salivary gland, and stomach. B3GNT3 is a member of GlcNAc transferases and part of glycobiology enzymes. Several studies have implicated glycobiology-related genes in autism spectrum disorders (Dwyer and Esko, 2016). Also, mutations in N acetylglucosaminyltransferase 1 have been associated with inherited forms of ASDs (Yuen et al., 2017).

Nevertheless, we found *de novo* variants in 15 genes that have been previously associated with or reported in patients with ASD or neurodevelopmental disorders. These include *PHF21A* (Kim et al., 2019), *WASF1* (Ito et al., 2018), *TCF20* (Vetrini et al., 2019), *DEAF1* (Rajab et al., 2015), *MED13* (Snijders Blok et al., 2018), *CREBBP* (Zheng et al., 2016), *KDM6B* (Stolerman et al., 2019), *SMURF1* (De Rubeis et al., 2014), *ADNP* (Helsmoortel et al., 2014), *CACNA1G* (Chemin et al., 2018), *MYT1L* (Blanchet et al., 2017), *KIF13B* (Chen et al., 2020), *GRIA2* (Salpietro et al., 2019), *CHM* (Tran et al., 2020), and *KCNK9* (Delgado et al., 2014). Our results are consistent with many previous reports. For example, we identified a *de novo* variant (c.215G>A/p.R72H, CADD score: 29.6) in *WASF1 in* an ASD subject who had epilepsy comorbidity from non-consanguineous parents. This variant was confirmed by Sanger sequencing (Figure 2).

Notably, a recent study has reported three nonsense *WASF1 de novo* mutations in five unrelated cases with Intellectual Disability and Seizures (Ito et al., 2018). Also, *WASF1* is considered a Rho family GTPases protein member, which is known to be involved in the pathogenesis of ASD (Guo et al., 2020). *ADNP* is another example of an essential and well-defined gene associated with ASD. Our study revealed a validated *de novo* variant in *ADNP* (c.2157C>A/p.Y719*, CADD score: 22.8), a gene in which multiple studies have reported variants in ASD patients (Helsmoortel et al., 2014; Vandeweyer et al., 2014; Rossi et al., 2017; Arnett et al., 2018). A study by Helsmoortel et al. (Helsmoortel et al., 2014) listed ten ASD patients with different clinical presentations, mainly ID and facial dysmorphism due to *ADNP* mutations. Besides, they estimated that nearly 1 in 10,000 ASD cases could have *ADNP* mutations. A recent *in vivo* study using *Adnp* knock-out mice showed a reduction in dendritic spine density and a change in gene expression due to Adnp deficiency (Hacohen-Kleiman et al., 2018). Our sequencing data revealed a deleterious *de novo* variant in *TCF20.* Consistent with our study, pathogenic variants in this gene have been reported in patients with ASD, intellectual impairment, behavioral abnormalities, and epilepsy (Babbs et al., 2014; Vetrini et al., 2019). Notably, about twenty-five inherited or *de novo* pathogenic variants in *TCF20* were recently observed in affected patients with clinical presentations similar to the phenotype observed in Smith–Magenis syndrome (Babbs et al., 2014; Vetrini et al., 2019). The novel *de novo* variant in *DEAF1* is predicted to be damaging and positioned in a highly conserved area of the genome and is not reported in the gnomAD database. A family-based study in a consanguineous Omani family using WES reported a homozygous variant in *DEAF1* in three affected siblings with autism and ID (Rajab et al., 2015). Also, recent studies have reported many deleterious *de novo* and recessive variants in *DEAF1* implicated with ASD genetic risk (Chen et al., 2017; Satterstrom et al., 2020).

Our study provides additional support for inherited genetic risk in ASD. We have identified three multiplex families with affected individuals who shared the same variants. One interesting case is a consanguineous family with identical triplets’ children (three monozygotic male siblings) who had ASD with variable clinical presentation, but all suffered from epilepsy. WES identified a homozygous missense variant in the *VPS13B* (c.8441G>A/p.R2814Q, CADD score: 32.0) in all three children (Table 3). Both parents were heterozygous for this variant, and all three ASD patients were homozygotes. The variant was infrequent in the gnomAD database (MAF = 0.00002, homozygous count = 0) and predicted to be pathogenic by functional prediction tools. Current evidence indicates a vital role for *VPS13B* in the normal growth and development of neurons. Pathogenic variants in *VPS13B* have been reported in patients with Cohen syndrome, a rare autosomal recessive disease characterized by intellectual disability, dysmorphism, and microcephaly (Yu et al., 2013). Sequencing results also revealed a homozygous missense variant in the *PIGN* (c.364G>C/p.E122Q, CADD score: 26.7) (Table 3) in the three ASD patients from this family. This mutation was rare in the gnomAD database (MAF = 0.0007, homozygous count = 1) and predicted to be pathogenic. The PIGN protein is involved in glycosylphosphatidylinositol (GPI)-anchor biosynthesis, and homozygous pathogenic variants in *PIGN* have been reported in patients with multiple congenital anomalies, including delayed psychomotor development, hypotonia, and seizures (Maydan et al., 2011; Tian et al., 2022). Our data reveal the complex genetic architecture of ASD and identify two homozygous variants in genes involved in neuronal development. Notably, expression of *VPS13B* and *PIGN* varies significantly during differentiation of wild-type iPSC to neurons suggesting a role for these two genes in neuronal development (Lizio et al., 2019). The variant in *PIGN* could explain the observed seizures in the patients, but further functional studies will be required to confirm this finding.

Also, our exome data presented another extended family with two affected boys (only one of them has seizures at 30 months) and one healthy sister. Interestingly, a homozygous missense variant in *C12orf57* (Table 3) was detected in both affected brothers but not in their healthy sister. This variant has been previously reported (c.A1G/p.M1V, CADD score: 21.9) in several consanguineous Saudi and Kuwaiti patients who displayed diverse clinical characteristics such as a profound global development delay, autistic features, and epilepsy (Akizu et al., 2013; Salih et al., 2013; Zahrani et al., 2013). Besides, *C12orf57* is associated with Temtamy syndrome comprised of hypotonia, moderate to severe ID with ASD features, corpus callosum hypoplasia, seizures, and microphthalmia (Akizu et al., 2013; Salih et al., 2013; Zahrani et al., 2013; Wang et al., 2019). Notably, our homozygosity mapping analysis showed that *C12orf57* falls within a 22 Mb run of homozygosity on chromosome 2 in both affected brothers. WES for the affected ASD patient with epilepsy revealed a deleterious X-linked variant in *WNK3* (c.236A>G/p.E79G, CADD score: 22.9) (Table 3) but this variant was not detected in his brother who had ASD without epilepsy. *WNK3* was reported to be partially deleted in two brothers with autism, ID, and cleft lip/palate and a maternally inherited microdeletion (Qiao et al., 2008). WNK3 protein was co-localized with Cl_2_ transporters (NKCC1 and KCC) and regulate the chloride (Cl_2_) ion transportation that revealed a critical role in the excitability of gamma-Aminobutyric acid (GABA) receptors in neurons (Kahle et al., 2005).

The third multiplex family includes two affected boys from a consanguineous marriage who share a likely pathogenic variant in *TSPYL2* (c.1668G>C/p.Q556H) on the X chromosome. *TSPYL2* was described in many patients with Xp11.2 microduplications and neurodevelopmental disorders (Moey et al., 2016). Furthermore, one affected individual had an additional rare variant (c.9565delG/p.V3189fs*7) in dynein axonemal heavy chain 3 (*DNAH3*) (Table 3). Loss of function variants in *DNAH3* has been described in large-scale studies of patients with ASD (the Simons Simplex Collection (Krumm et al., 2015) and in a Chinese study (Guo et al., 2017)).

Collectively, the evidence from this study suggests that 75% of multiplex families (6 out of 8) showed at least two siblings sharing the same variants. This is higher than previous studies (that reported about 30% of affected siblings carrying same variants relevant to ASD risk) which is likely due to the prevalent consanguinity. Besides, there were novel genetic findings that have an additional individual risk effect to ASD.

Furthermore, data from one ASD trio showed a likely pathogenic homozygous variant in *CADPS2*. The identified variant was in a highly conserved genomic area with no recorded allele frequencies in gnomAD or dbSNP databases. This gene is located in the 7q31 region that is has been reported to be deleted in patients with ASD (Okamoto et al., 2011; Grabowski et al., 2017). Studies using animal models showed that *Cadps2*-knockout mice exhibited a brain impairment and autistic-like behavioral phenotypes (Sadakata et al., 2007). Besides, *CADPS2* variant screening in Italian patients with ID and with ASD subjects have shown parental inheritance for deleterious variants with a suggestion of parent-of-origin effect (Bonora et al., 2014).

It is well known that ASD complexities may be subjected to the multiple heterogeneity threshold or dosage (Schaaf et al., 2011; Cristina et al., 2013), where affected subjects may inherit different genetic risks (either novel or *de novo* mutations) in known genes, as well as chromosomal anomalies (CNV). Together these genetic risks may contribute as additive interacting factors to ASD-threshold (Cook and Scherer, 2008). Significantly, we recognized such multifactorial effects in several families (Table 4). For instance, one simplex family had variants in two candidate genes; a *de novo* p.G727R in *SMURF1* which was validated by Sanger Sequencing (Supplementary Figure S1), and a recessive p.P635L in *NAALAD2*. *SMURF1* encodes a ubiquitin ligase protein that is essential as a regulatory receptor for the SMAD proteins in the bone morphogenetic protein (BMP) pathway (Cui et al., 2011). Also, SMURF1 was found to play a role in controlling cell motility, signaling, and polarity (Zhu et al., 1999). Moreover, *SMURF1* was significantly associated with ASD risk as a *de novo* splice site variant, as recently described (De Rubeis et al., 2014) by the Autism Sequencing Consortium patient in the Hartwell Autism Research and Technology Initiative (iHART) cohort (Ruzzo et al., 2019). Regarding the *NAALAD2* gene, the NAALAD2 protein is considered one of the N-acetylated alpha-linked acidic dipeptidase (NAALADase) gene families. NAALADase was proposed to have an associated role with N-acetyl-L-aspartate-L-glutamate (NAAG) in the central nervous systems. But there is no clear link between NAALAD2 and ASD or any neurodevelopmental disorders (Pangalos et al., 1999).

Another example of oligo-heterogeneity was observed in a consanguineous family with a child affected with ASD and early onset epilepsy (9 months). The WES data identified a *de novo* variant in *KCNK9* (c.479T>C/p.V160A, CADD score: 25.7) (Table 4). *KCNK9* (potassium channel subfamily K member 9) is a channelopathy protein associated with Birk-Barel syndrome. This gene is imprinted with paternal silencing, and the mutation entirely abolishes the potassium channel activity when acting as a homodimer and when functioning as a heterodimer (Barel et al., 2008). Two recent studies have described patients with developmental delay and central hypotonia with *de novo* variants in *KCNK9* by exome sequencing (Graham et al., 2016; Šedivá et al., 2020). Nevertheless, the child had also a likely pathogenic homozygous variant in *TRAF3IP1* (c.1064G>A/p.G355E, CADD score: 29.6). The variant is rare, with reported allele frequencies of 0.01% in gnomAD and 0.02% in the 1,000 genomes. *TRAF3IP1* is an autosomal recessive ciliopathy gene associated with Senior-Loken syndrome nine that is represented by early-onset nephronophthisis and pigmentary retinopathy (Bizet et al., 2015). Besides, a study reported five consanguineous families with four homozygous recessive and one compound heterozygous mutations using exome sequencing, where few families had affected subjects with global developmental delay and severe cognitive impairment.(Bizet et al., 2015).

Our study also identified a novel missense mutation in *SUOX* (in case 33; c.1156G>A/p.V386M, CADD score: 26.7 Supplementary Table S4). This gene encodes an enzyme, Sulfite oxidase, that catalyzes the oxidation of sulfite to sulfate (Irreverre et al., 1967). The deficiency of this enzyme has been associated with hereditary metabolic disorders and lethal neurological complications that occur at an early age (Rupar et al., 1996). Nevertheless, several previously published variants of *SUOX* were reported in patients with seizures, feeding difficulties, microcephaly, and brain atrophy.(Rupar et al., 1996; Johnson et al., 2002). Further work needs to be done, including the follow-up of the subject with this identified inborn metabolic errors for re-examining and the testing of sulfite oxidase deficiency or other metabolic profiling.

This study highlighted many genes that warrant further investigation. Future studies should aim at functional studies to characterize the identified genes deeply using animal models and human-induced pluripotent stem cells (hiPSC). The functional studies for the identified putative ASD genes using the known animal models in mice (Ergaz et al., 2016), *Drosophila* (Bellosta and Soldano, 2019), or zebrafish (Meshalkina et al., 2018); will help us understand the etiology, pathogenesis, and treatment of human ASD. Furthermore, taking advantage of patient-derived stem cell research, the researchers are now using the human-induced potent stem cell technology to generate neurons *in vitro* to model ASDs (Russo et al., 2019; Pensado-López et al., 2020).

We are aware that our project has limitations since our analysis focused on the coding regions for most cases. These constraints were due to the sequencing-platform variabilities. Another limitation of this study is that it was not possible to validate all identified *de novo* mutations using Sanger sequencing. However, all those picked variants with a minimum depth coverage were validated. Additionally, the combined sequencing depth for most of the remaining mutations was very high.

In summary, in our study, the known syndromic neurodevelopmental disorders indicated that ∼7% (7/102) were similar to the reported contribution of classical ASD etiology (1%–5%). Additionally, we identified ∼15% (15/102) of the ASD families due to possessing potential CNV regions. On the other hand, our study showed that 44.32% (39 out of 88 ASD cases) of studied cases had variants in genes that are relevant to ASD or related neurodevelopmental disorders. This finding is consistent with the power of clinical NGS as a diagnostic tool for neurodevelopmental (Álvarez-Mora et al., 2022). However, this rate is slightly higher than what has been reported previously for ASD (Stefanski et al., 2021) because it was estimated for genes related to ASD from the literature and the SAFARI database. However, some of these genes warrant functional studies to confirm their involvement in ASD. After crossing the reported genes in this study with the SAFARI database and the recent large SPARK study (Zhou et al., 2022), the rate was approximately ∼28% (25/88).

Furthermore, we crossed our findings with the most recent study from the region that shared a similar scope to our project (Abdi et al., 2023). Regarding the patient background, our cohort had a broader representation of the Middle East and North Africa, with ∼89% *versus* approximately 50% of Arab in Abdi et al. study. There was no overlap in top candidate variants between the two studies which is expected in highly heterogenous disorders such as ASD. Our study has revealed a significant homozygous burden within our ASD cohort, with an estimated rate of 63% (12/19) among consanguineous ASD cases and 12% (2/17) among non-consanguineous cases. Notably, Our findings are in alignment with the homozygous burden observed in consanguineous *versus* non-consanguineous families in the Abdi et al. Study.

Our data have revealed several genetic factors associated with either known or rare syndromes that have neurodevelopmental impacts only or have multiple body system effects combining with the nervous system (Birk-Barel syndrome (*KCNK9*), Temtamy syndrome (*C12orf57*), Rubinstein-Taybi syndrome 1 (*CREBBP*), Chediak-Higashi syndrome (*LYST*), Deafness (*TECTA*), Cohen syndrome (*VPS13B*), Escobar syndrome (*CHRNG*), and Arrhythmogenic right ventricular dysplasia 2 (*RYR2*)). We acknowledge that in our study, the information provided to our research team was limited to ASD-positive cases and the reported consanguinity within families. Although we recognize the importance of comprehensive clinical characterization, the constraints on available clinical data necessitated our reliance on standardized assessment tools and limited information provided by the families. However, the replication of these findings resulted from various applied molecular genetic testing, which showed high confidence in explaining the multifactorial condition of ASD.

Finally, despite the ASD multifactorial etiology that complicates the discovery of ASD risk variants, the number of identified novel or known putative ASD genes from our study was appreciable. Moreover, this study represents the first large-scale characterization of the ASD genetic risk in Qatar’s Middle Eastern population.

## Data Availability

The datasets presented in this article are not readily accessible as the data is protected and inaccessible due to privacy regulations that aim to safeguard the privacy and consent of research participants. However, the data that supports the findings of this study is available upon request from the corresponding author [YA-S and OA].

## Abbreviations

ASD, Autism spectrum disorder; ID, Intellectual Disability; ADHD, Attention Deficit Hyperactivity Disorder; DSM-V, Diagnostic and Statistical Manual of Mental Disorders; CNV, Copy Number Variations; GWAS, Genome-Wide Association Studies; SNP, Single Nucleotide Polymorphisms; ADI-R, The Autism Diagnostic Interview-Revised; ADOS, Autism Diagnostic Observation Schedule; IQ, Intelligence Quotient; IRB, Institutional Review Board; MLPA, Multiplex Ligation dependent Probe Amplification; WES, Whole-Exome Sequencing; WGS, Whole-genome Sequencing; QCII, QIAGEN Clinical Insight Interpret.
