# Supplementary material for: The genetic landscape of autism spectrum disorder in the Middle Eastern population
Source: Front Genet. 2024 Mar 20;15:1363849. doi: 10.3389/fgene.2024.1363849 (PMC10987745; doi:10.3389/fgene.2024.1363849)
Supplement: Supplementary file 1 [file DataSheet1.docx]

**Supplementary Materials:**

**Table S1:** Summary of CNVs detected in ASD patients.

**Table S2**: *De novo* variants in ASD patients detected in genes with no substantial evidence of involvement in ASD*.*

**Table S3**: Trio-combined depth coverage for the identified *de novo* variants.

**Table S4:** Recessive variations in ASD patients detected in genes with no substantial evidence of involvement in ASD.

**Table S5:** QIAGEN Clinical Insight Interpret version 9.0.0.20220826 database freeze list.

**Figure S1:** Identified CNV regions in Case 18, Case 11, and Case 16.

**Figure S2:** Validation of *SMURF1* *de novo* variation by Sanger sequencing.

**Figure S3:** Validation of *CACNA1G* *de novo* variation by Sanger sequencing.

**Figure S4:** Validation of *B3GNT3 de novo* variation by Sanger sequencing.

**Supplementary Table S1:** Summary of CNVs detected in ASD patients.

| **Subject ID** | **CNV** | **Size (Mb)** | **Inheritance** | **DECIPHER-Patient variants and clinical characterization*** |
| --- | --- | --- | --- | --- |
| 1 | Loss chr2:96737083-98202258 | 1.47 | Parental | CNV loss 8 out of 52, including de novo, paternally inherited heterozygous pathogenic deletion size ranged (1Mb to 25 Mb), associated commonly with Autism, Global developmental delay, and congenital anomalies. |
| 2 | Gain chr19:27862694-28769462 | 0.91 | Parental | CNV gain 6 out of 33, including de novo, paternally inherited heterozygous pathogenic duplication size ranged (359 KB to 21 Mb), associated commonly with Autism, Global developmental delay, and Delayed speech and language development |
| 3 | Gain chr8:42935861-43831881 | 0.90 | Parental | CNV gain 15 out of 62, including de novo, paternally inherited heterozygous pathogenic duplication size ranged (3.9Mb to 144.5 Mb), associated commonly with Autism, Global developmental delay, and Intellectual Disability |
| 4 | Gain chr8:42936890-43831881 | 0.89 | Parental | CNV gain 15 out of 62, including de novo, paternally inherited heterozygous pathogenic duplication size ranged (3.9Mb to 144.5 Mb), associated commonly with Autism, Global developmental delay, and Intellectual Disability |
| 5 | Loss chr2:10416994-12516176 | 2.10 | *De novo* | CNV loss 16 out of 59, including de novo, paternally inherited heterozygous pathogenic deletion size ranged (1.5Mb to 25 Mb), associated commonly with Global developmental delay and congenital anomalies. |
| 6 | Gain chr5:103170089-103901164 | 0.73 | Parental | CNV gain 5 out of 32, including de novo, paternally inherited heterozygous pathogenic duplication size ranged (17Mb to 20Mb), associated commonly with Autistic behavior, Behavioral abnormality, Microcephaly, Single transverse palmar crease, Specific learning disability. |
| 7 | Gain chr7:62043624-62650353 | 0.61 | Maternal | CNV gain 4 out of 15, including de novo inherited heterozygous pathogenic duplication size ranged (20.9 Mb to 23Mb), associated commonly with Intellectual Disability, Microcephaly, and Delayed speech and language development. |
| 8 | Loss chr15:28969665-30371774 | 1.40 | Maternal | CNV loss 63 out of 254, including de novo inherited heterozygous pathogenic deletion size ranged (1.3 Mb to 16Mb), associated commonly with Ataxia, Intellectual disability, and Autism. |

* annotation described in the DECIPHER database related to clinical presentations of ASD[146]

**Supplementary Table S1.** (Continued) Summary of CNVs detected in ASD patients

| **Subject ID** | **CNV** | **Size (Mb)** | **Inheritance** | **DECIPHER-Patient variants and clinical characterization*** |
| --- | --- | --- | --- | --- |
| 9 | Loss  chr22:18846330-20312668 | 1.47 | *De novo* | CNV loss 345 out of 919, including paternally inherited heterozygous pathogenic deletion size ranged (21.5 Kb to 9.2 Mb), associated commonly with Autistic behavior, Delayed speech and language development, and Intellectual disability. |
| 10 | Gain  chr15:22753733-23367070 | 0.61 | Maternal | CNV gain 64 out of 60, including de novo, paternally inherited heterozygous pathogenic duplication size ranged (253.9 kb to 79.5 Mb), associated commonly with Autism, Global developmental delay, and Intellectual Disability. |
| 11 | Loss  chr15:84931022-85728834 | 0.80 | Maternal | CNV loss 8 out of 75, including de novo, paternally inherited heterozygous pathogenic deletion size ranged (718 kb to 24 Mb), associated commonly with Intellectual Disability and Delayed speech and language development. |
| 12 | Loss  chr9:91126991-101328200 | 10.20 | Unknown** | CNV loss 8 out of 75, including de novo, paternally inherited heterozygous pathogenic deletion size ranged (152 kb to 15 Mb), associated commonly with Intellectual Disability, Autistic behavior, Global developmental delay, and Delayed speech and language development. |
| 13 | Gain chr13:113044023-113727674 | 0.68 | Denovo | CNV gain 17 out of 162, including de novo, paternally inherited heterozygous pathogenic duplication size ranged (241 kb to 96 Mb), associated commonly with congenital anomalies, Global developmental delay, and Intellectual Disability. |
| 14 | Gain  chr7:16839835-17769420 | 0.93 | Maternal | CNV gain 15 out of 80, including de novo, paternally inherited heterozygous pathogenic duplication size ranged (253 kb to 20.8 Mb), associated commonly with congenital anomalies, Intellectual Disability, and Global developmental delay. |
| 15 | Loss  chr22:18889490-20312668 | 1.42 | Unknown** | CNV loss 345 out of 917, including de novo, paternally inherited heterozygous pathogenic deletion size ranged (21 kb to 9.2 Mb), associated commonly with Intellectual Disability, Autism, and Global developmental delay. |

* annotation described in the DECIPHER database related to clinical presentations of ASD[146]

** Unknown, the region was not inherited from the mother, and the father genotype was missing.

**Supplementary Table S1.** (Continued) Summary of CNVs detected in ASD patients.

| **Subject ID** | **CNV** | **Size (Mb)** | **Inheritance** | **DECIPHER-Patient variants and clinical characterization*** |
| --- | --- | --- | --- | --- |
| 16 | Gain  chr1:174802731-175908200 | 1.11 | Maternal | CNV gain 3 out of 54, including Unknown inherited heterozygous pathogenic duplication size ranged (308 kb to 6.5 Mb), associated commonly with abnormal facial shape and growth delay. |
| 16 | Gain  chr8:42925966-43831881 | 0.91 | Maternal | CNV gain 15 out of 62, including de novo, paternally inherited heterozygous pathogenic duplication size ranged (3.9 Mb to 144.9 Mb), associated commonly with Autism and Global Developmental Delay. |

* annotation described in the DECIPHER database related to clinical presentations of ASD [146].

** Unknown, the region was not inherited from the mother, and the father genotype was missing.

**Supplementary Table S2**: *De novo variants in ASD patients detected in genes with no substantial evidence of involvement in ASD.*

| **Sample ID** | **Protein** | **Gene** | **CADD Score** | **dbSNP ID*** | **gnomAD Frequency****^%^ | **gnomAD Homozygous Count** | **^***^ SFARI Genes Score** |
| --- | --- | --- | --- | --- | --- | --- | --- |
| 2 | p.K87E | *DEPTOR* | 22.20 | NA | NA | NA | NA |
| 9 | p.P8L | *PRR23A* | 23.40 | NA | NA | NA | NA |
| 10 | p.D891N | *ADAMTS9* | 22.30 | 554151476 | 0.003 | 0 | NA |
| 23 | p.Q99L | *MAP3K10* | 21.90 | NA | NA | NA | NA |
| 25 | p.R835C | *ATAD5* | 29.10 | 143854673 | 0.003 | 0 | NA |
| 26 | c.632+5G>C^ | *SCAMP2* | 25.10 | NA | NA | NA | NA |
| 39 | p.G260D | *GALC* | 26.00 | 199847983 | 0.002 | 0 | NA |
| 41 | p.L150* | *MRTFA* | NA | NA | NA | NA | NA |
| 42 | p.A1098_G1108del | *FMN2* | NA | NA | NA | NA | NA |
| 44 | p.V581G | *SP3* | 22.80 | NA | NA | NA | NA |
| 51 | p.L18_G23dup | *SF1* | NA | 1208325707 | 0 | 0 | NA |
| 54 | p.D116G | *AKAP6* | 27.60 | NA | NA | NA | NA |
| 55 | p.V223D | *CAPG* | 29.90 | NA | NA | NA | NA |

A summary of the additional *de novo* variants identified in ASD patients occurring in genes with no substantial evidence of involvement in ASD or neurodevelopmental disorders. Identified variant(s) per family separated by borderline. ^Coding DNA reference sequences reported with Promoter Loss or Splice Site Loss. * NA indicates novel variants not previously reported in the gnomAD or SFARI databases. ** Reported gnomAD frequency was observed out of 141,456 individuals. **^***^** SFARI Gene Score is a ranking system that estimates the strength of evidence of the reported ASD gene in the SFARI database, with S= ASD syndromic category and scores ranging from 1 to 3, with 1 being the higher score indicating the stronger association evidence with ASD**.**

**Table S3**: Trio-combined depth coverage for the identified *de novo* variants.

| **Sample ID** | **Protein** | **Gene** | **Child DP** | **Mother DP** | **Father DP** | **Trios DP** |
| --- | --- | --- | --- | --- | --- | --- |
| 2 | p.K87E | *DEPTOR* | 192 | 57 | 130 | 379 |
| 3 | p.K468T | *DTX4* | 29 | 27 | 34 | 90 |
| 4 | p.P320R | *NLRP5* | 413 | 97 | 52 | 562 |
| 9 | p.P8L | *PRR23A* | 102 | 106 | 195 | 403 |
| 10 | p.D891N | *ADAMTS9* | 29 | 40 | 39 | 108 |
| 12 | p.A131V | *ARMC6* | 16 | 35 | 21 | 72 |
| 14 | p.T469I | *DRD5* | 98 | 51 | 97 | 246 |
| 16 | p.P399Q | *PHF21A* | 106 | 54 | 38 | 198 |
| **19** | **p.R72H** | ***WASF1*** | **30** | **WT** | **WT** | **30** |
| 23 | p.Q99L | *MAP3K10* | 577 | 40 | 37 | 654 |
| 24 | p.P1942R | *TCF20* | 110 | 36 | 42 | 188 |
| 25 | p.D9G | *DEAF1* | 87 | 89 | 52 | 228 |
| 25 | p.R835C | *ATAD5* | 78 | 100 | 57 | 235 |
| 26 | c.632+5G>C^ | *SCAMP2* | 64 | 45 | 38 | 147 |
| 26 | p.V346I | *MED13* | 80 | 37 | 39 | 156 |
| 27 | p.H1432R | *CREBBP* | 107 | 65 | 64 | 236 |
| 27 | c.870-10T>G^ | *SETD1A* | 67 | 37 | 36 | 140 |
| 28 | p.S1198L | *KDM6B* | 246 | 33 | 38 | 317 |
| **34** | **p.G724R** | ***SMURF1*** | **62** | **WT** | **WT** | **62** |
| 35 | p.D396G | *ABCA2* | 50 | 65 | 64 | 179 |
| **36** | **p.Y719*** | ***ADNP*** | **37** | **WT** | **WT** | **37** |
| **38** | **p.E11*** | ***CACNA1G*** | **13** | **WT** | **WT** | **13** |
| **38** | **p.A167V** | ***B3GNT3*** | **28** | **WT** | **WT** | **28** |
| 39 | p.G260D | *GALC* | 11 | WT | WT | 11 |
| 40 | p.V13A | *CHM* | 41 | WT | WT | 41 |
| 41 | p.L150* | *MRTFA* | 79 | 35 | 33 | 147 |
| 42 | p.A1098_G1108del | *FMN2* | 31 | 29 | 79 | 139 |
| 43 | p.G573R | *MYT1L* | 201 | 71 | 70 | 342 |
| 44 | p.V581G | *SP3* | 53 | 44 | 40 | 137 |
| 45 | p.R1086* | *KIF13B* | 38 | 49 | 26 | 113 |
| 46 | p.W580C | *GRIA2* | 42 | 35 | 41 | 118 |
| 47 | p.V160A | *KCNK9* | 159 | 50 | 57 | 266 |
| 48 | p.R458C; | *CHRNG* | 196 | 71 | 35 | 302 |
| 51 | p.L18_G23dup | *SF1* | 87 | 70 | 39 | 196 |
| 52 | p.K337R | *KDM2A* | 170 | 137 | 78 | 385 |
| 54 | p.D116G | *AKAP6* | 133 | 87 | 96 | 316 |
| 55 | p.V223D | *CAPG* | 91 | 37 | 40 | 168 |

DP is the total depth of coverage. Identified variant(s) per family separated by borderline. The following criteria were applied to exclude false-positive results for de novo variants: A combined sequencing depth for the trio (DP≥30) and a minimum of individual depth (DP≥10). In addition, only *de novo* variations with allele fraction of 35-65% in each subject were considered. WT: Wilde type reference allele. Genes indicated in bold are validated and confirmed by Sanger sequencing among the trio families.

**Supplementary Table S4:** Recessive variations in ASD patients detected in genes with no substantial evidence of involvement in ASD.

| **Sample ID** | **Protein** | **Gene** | **CADD Score** | **dbSNP ID** | **gnomAD Frequency^*^** | **gnomAD Homozygous Count** | **^***^ SFARI Genes Score** |
| --- | --- | --- | --- | --- | --- | --- | --- |
| 1 | c.354+2 T>G | *NAPB^$^* | 33 | NA | NA | NA | NA |
| 5 | p.R310C | *TG* | 26.8 | 555719562 | 0.047 | 1 | NA |
| 5 | p.R734H | *GBA2* | 23.1 | 142621039 | 0.064 | 1 | NA |
| 7 | p.G154E | *PRSS57* | 20.2 | 1360346872 | 0 | 0 | NA |
| 11 | p.R273C | *ADH1B* | 24.7 | 760179023 | 0.009 | 0 | NA |
| 11 | p.K240Q | *KRT73* | 24.9 | 867784027 | NA | NA | NA |
| 12 | p.I406T | *CERKL* | 27.7 | 141723283 | 0.006 | 0 | NA |
| 12 | p.Y846* | *ANKRD62* | 36 | NA | NA | NA | NA |
| 12 | p.R731W | *ZNF236* | 24.5 | NA | NA | NA | NA |
| 13 | p.G343R | *RGS3* | 35 | 201562824 | 0.031 | 0 | NA |
| 17 | p.L777F | *PXDNL* | 22.4 | 759113039 | 0.007 | 0 | NA |
| 20 | p.P53L | *CBY1* | 24.2 | 199883977 | 0.012 | 0 | NA |
| 22 | p.R47P | *CLIC3* | 23 | NA | NA | NA | NA |
| 29 | p.R91H | *JAKMIP3* | 29 | 753880520 | 0.001 | 0 | NA |
| 30 | p.N457fs*26 | *SPICE1* | NA | NA | NA | NA | NA |
| 31 | p.R427C | *CNGA3* | 29.8 | 141386891 | 0.039 | 1 | NA |
| 31 | p.I165T | *DKK4* | 26.9 | 541376818 | 0.072 | 0 | NA |
| 31 | p.G439R | *ENTPD2* | 28.7 | 781654598 | 0.008 | 0 | NA |
| 32 | p.R698H | *EPHA8* | 20.5 | 758537106 | 0.001 | 0 | NA |
| 32 | p.R240Q | *HEYL* | 25.3 | 995053866 | 0.001 | 0 | NA |
| 32 | p.I200fs*14 | *PI16* | NA | NA | NA | NA | NA |
| 32 | c.-129G>T^ | *CDCA2* | 23.3 | 145029103 | NA | NA | NA |
| 32 | p.S1359A | *LAMA3* | 25.1 | 767046193 | 0.003 | 0 | NA |

A summary of the additional recessive variants identified in ASD patients occurring in genes with no substantial evidence of involvement in ASD or neurodevelopmental disorders. Identified variant(s) per family separated by borderline. ^Coding DNA reference sequences reported with Promoter Loss or Splice Site Loss. * NA indicates novel variants not previously reported in the gnomAD or SFARI databases. ** Reported gnomAD frequency was observed out of 141,456 individuals. **^***^** SFARI Gene Score is a ranking system that estimates the strength of evidence of the reported ASD gene in the SFARI database, with S= ASD syndromic category and scores ranging from 1 to 3, with 1 being the higher score indicating the stronger association evidence with ASD**. ^$^**Gene Associated with Developmental and epileptic encephalopathy.

**Supplementary Table S4:** (Continued) Recessive variations in ASD patients detected in genes with no substantial evidence of involvement in ASD.

| **Sample ID** | **Protein** | **Gene** | **CADD Score** | **dbSNP ID** | **gnomAD Frequency^**%^** | **gnomAD Homozygous Count** | **^***^ SFARI Genes Score** |
| --- | --- | --- | --- | --- | --- | --- | --- |
| 33 | p.S901C | *NLRP14* | 25.3 | 867723894 | 0.003 | 0 | NA |
| 33 | p.M21I | *HOXC5* | 27 | 1191702346 | 0 | 0 | NA |
| 33 | p.V386M | *SUOX* | 26.7 | 775888022 | NA | NA | NA |
| 34 | p.P280S | *NAALAD2* | 26.1 | 200163147 | 0.012 | 0 | NA |
| 37 | p.R338C | *SPN* | 23.6 | 200681097 | 0.029 | 1 | NA |
| 38 | p.K884N | *DNAH14* | 22.8 | 570373884 | 0.036 | 0 | NA |
| 38 | p.D331N | *PGBD4* | 23.4 | 146189712 | 0.002 | 0 | NA |
| 38 | p.R493L | *GTSE1* | 23.4 | 201709046 | 0.014 | 0 | NA |
| 38 | p.S69T | *IL1RAPL2* | 23.9 | NA | NA | NA | NA |
| 41 | p.T973_P984del | *WNK2* | NA | 1354506209 | 0.000007 | NA | NA |
| 47 | p.G355E | *TRAF3IP1* | 29.6 | 560016209 | 0.012 | 0 | NA |
| 47 | p.K241* | *C1QTNF4* | 39 | NA | NA | NA | NA |
| 47 | p.F329C | *SLCO1B7* | 21.5 | 201253485 | 0.043 | 1 | NA |
| 47 | p.R29C | *NPTX1* | 28.8 | NA | NA | NA | NA |
| 54 | p.L440Q | *PCDHGA3* | 22.7 | 759545017 | 0.005 | 0 | NA |
| 54 | p.R929C | *PCDH12* | 26.3 | 182860036 | 0.004 | 0 | NA |
| 54 | p.R193* | *PCK2* | 38 | 753706965 | 0.008 | 0 | NA |
| 54 | p.G171R | *SLC25A21* | 31 | 145395890 | 0.091 | 2 | NA |
| 55 | c.354+2 T>G | *NAPB***^$^** | 33 | NA | NA | NA | NA |
| 56 | c.354+2 T>G | *NAPB***^$^** | 33 | NA | NA | NA | NA |

A summary of the additional recessive variants identified in ASD patients occurs in genes with no substantial evidence of involvement in ASD or neurodevelopmental disorders. Identified variant(s) per family separated by borderline. ^Coding DNA reference sequences reported with Promoter Loss or Splice Site Loss. * NA indicates novel variants not previously reported in the gnomAD or SFARI databases. ** Reported gnomAD frequency was observed out of 141,456 individuals. **^***^** SFARI Gene Score is a ranking system that estimates the strength of evidence of the reported ASD gene in the SFARI database, with scores ranging from 1 to 3, with 1 being the higher score indicating the stronger association evidence with ASD. **^$^**Gene Associated with Developmental and epileptic encephalopathy.

**Supplementary Table S5:** QIAGEN Clinical Insight Interpret version 9.0.0.20220826 database freeze list.

| **Content** | **Versions** |
| --- | --- |
| CADD | (v1.6) |
| Allele Frequency Community | (2019-09-25) |
| EVS | (ESP6500SI-V2) |
| Refseq Gene Model | (2020-04-06) |
| JASPAR | (2013-11) |
| Ingenuity Knowledge Base | (2022-01-29) |
| Vista Enhancer | (2012-07) |
| Clinical Trials: (B-release) | (B-release) |
| MITOMAP: A Human Mitochondrial Genome Database | (2020-06-19) |
| PolyPhen-2 | (v2.2.2) |
| 1000 Genome Frequency | (phase3v5b) |
| ExAC (0.3.1) | (0.3.1) |
| TargetScan | (7.2) |
| phyloP (NCBI36 (hg18) | (2009-11) |
| phyloP (GRCh37 (hg19) | (2014-02) |
| phyloP (GRCh38) | (2015-05) |
| GENCODE | (Release 33) |
| CentoMD | (5.3) |
| Ingenuity Knowledge Base | (B-release) |
| OMIM | OMIM |
| gnomAD (2.1.1) | (2.1.1) |
| BSIFT | (2016-02-23) |
| TCGA | (2013-09-05) |
| Clinvar | (2020-09-15) |
| DGV | (2016-05-15) |
| COSMIC | (v92) |
| HGMD (2022.4) | (2020.4) |
| OncoTree | (oncotree_2019_03_01) |
| dbSNP (NCBI36) | (dbSNP 151) |
| dbSNP (GRCh37) | (dbSNP 153) |
| dbSNP (GRCh38) | (dbSNP 153) |
| SIFT4G | (2016-02-23) |

**Supplementary Figure S1:** Identified CNV regions in Case 18, Case 11, and Case 16.

CNV region presenting the B Allele Freq, Log R Ratio, and CNV plots. Breakpoint indicated with red borders. The CNV region is compared and aligned between the Proband, Mother, and Father.

**Supplementary Figure S2:** Validation of *SMURF1* *de novo* variation by Sanger sequencing.

Sanger sequencing results for case34 with *SMURF1* variation (c.2170G>A/p.G724R). Box indicates de novo alteration (in the child) and wild-type allele (in parents).

**Supplementary Figure S3:** Validation of *CACNA1G* *de novo* variation by Sanger sequencing.

Sanger sequencing results for case38 with *CACNA1G* variation (c.31G>T/p.E11*). Box indicates de novo alteration (in the child) and wild-type allele (in parents).

******

**Supplementary Figure S4:** Validation of *B3GNT3* *de novo* variation by Sanger sequencing.

Sanger sequencing results for case38 with *B3GNT3* variation (c.500C>T/p.A167V). Box indicates de novo alteration (in the child) and wild-type allele (in parents).
